# Bioactivity of volatile organic compounds produced by *Pseudomonas tolaasii*

**DOI:** 10.3389/fmicb.2015.01082

**Published:** 2015-10-06

**Authors:** Pietro Lo Cantore, Annalisa Giorgio, Nicola S. Iacobellis

**Affiliations:** Scuola di Scienze Agrarie, Forestali, Alimentari e Ambientali, Università degli Studi della BasilicataPotenza, Italy

**Keywords:** *Pseudomonas tolaasii*, mushrooms, broccoli, lettuce, volatile organic compounds, methanethiol, dimethyl disulfide, 1-undecene

## Abstract

*Pseudomonas tolaasii* is the main bacterial pathogen of several mushroom species. In this paper we report that strains of *P. tolaasii* produce volatile substances inducing *in vitro* mycelia growth inhibition of *Pleurotus ostreatus* and *P. eryngii*, and *Agaricus bisporus* and *P. ostreatus* basidiome tissue blocks brown discoloration. *P. tolaasii* strains produced the volatile ammonia but not hydrogen cyanide. Among the volatiles detected by GC–MS, methanethiol, dimethyl disulfide (DMDS), and 1-undecene were identified. The latter, when assayed individually as pure compounds, led to similar effects noticed when *P. tolaasii* volatiles natural blend was used on mushrooms mycelia and basidiome tissue blocks. Furthermore, the natural volatile mixture resulted toxic toward lettuce and broccoli seedling growth. In contrast, pure volatiles showed different activity according to their nature and/or doses applied. Indeed, methanethiol resulted toxic at all the doses used, while DMDS toxicity was assessed till a quantity of 1.25 μg, below which it caused, together with 1-undecene (≥10 μg), broccoli growth increase.

## Introduction

*Pseudomonas tolaasii* (Paine, 1919) is the brown blotch disease’s causal agent in several economically important edible mushrooms such as *Agaricus bisporus* (Lange) Imbach (Tolaas, 1915), *A. bitorquis* (Quél.) Sacc. and other *Agaricales* (Gandy, 1979), *Pleurotus ostreatus* (Jacq. : Fr.) Kum (Gill, 1995), *P. eryngii* (D. C. : Fr.) Quél (Ferri, 1985; Rodriguez and Royse, 2007), *Lentinula edodes* (Berkeley) Singer (Tsuneda et al., 1995), and *Flammulina velutipes* (Curtis) Singer (Lee et al., 1996). Brown blotch is considered to be the cultivated mushrooms’s main disease because of the important economic losses (Fermor, 1987; Iacobellis, 2011) and the difficulty to control it. *C*ompost and casing soil, required for mushroom cultivation were found as a primary source of *P. tolaasii* (Wong and Preece, 1980).

*Pseudomonas tolaasii* is also described as a pathogen of some plants, indeed it causes diseases on cauliflowers (*Brassica oleracea* L. var. *botrytis*) (Talame and Piccirillo, 1995), tobacco (*Nicotiana tabacum* L.), *Solanum* sp. (Rainey et al., 1991; Shirata et al., 1995) and strawberry (*Fragaria* ×* ananassa* Duchesne) (Tanprasert and Reed, 1997). Moreover, it has been reported as a saprophytic bacterium associated to pear (*Pyrus communis* L.) phylloplane (Noval et al., 1993) and bean (*Phaseolus vulgaris* L.) and sugar beet (*Beta vulgaris* L.) rhizosphere (Zdor and Anderson, 1992).

*Pseudomonas tolaasii* produces tolaasins, considered the primary factors involved in the virulence and interaction between the pathogen and the hosts (Rainey et al., 1992; Soler-Rivas et al., 1999; Largeteau and Savoie, 2010; Iacobellis, 2011). The toxic lipodepsipeptides tolaasins (Nutkins et al., 1991) are the only molecules whose role in the virulence of *P. tolaasii* has been unequivocally ascertained (Cutri et al., 1984; Rainey et al., 1992; Grewal et al., 1995; Lo Cantore et al., 2006). In fact, when applied directly on mushrooms, tolaasins can reproduce the symptoms of the disease.

Besides tolaasins, other compounds have been reported to be produced by *P. tolaasii* and considered as potential factors responsible for bacterial botch symptoms. Park et al. (1994) isolated a compound from a *P. tolaasii* strain, characterized as an aminobenzene, containing an amylamine group, able to induce the symptoms of the disease on *A. bisporus* caps. Furthermore, Shirata (1996) described the ability of *P. tolaasii* strains to produce volatile organic compounds (VOCs) called tovsins, different from tolaasins, which are not volatiles, and able to induce the alteration of *P. ostreatus* and *F. velutipes* basidiomes.

Volatile organic compounds are small molecules (molecular masses lower 300 Da) with low polarity and high vapor pressure that are produced by both eukaryotes and prokaryotes. The biological activities and role of many bacterial VOCs, so far identified, are partly known; in fact, they have been demonstrated to act as infochemicals for inter- and intra-organism communication (Effmert et al., 2012), attraction, and defense (Ryu et al., 2004; Farag et al., 2006). They can be detected in small amounts by the organisms and diffused in the atmosphere and soil (Kai et al., 2009; Morath et al., 2012).

The bacterial volatile blends have been proved to interact with plants and fungi by inhibiting or stimulating their growth (Kai et al., 2007; Vespermann et al., 2007; Effmert et al., 2012). To date, many studies have been performed with the aim of identifying bacterial volatile substances, including those produced by *Pseudomonas* sp.. However, the knowledge on their biological activities is still limited. In fact, only in recent years the inhibition and/or stimulation of fungal and plant growth by carbon dioxide (CO_2_), hydrogen cyanide (HCN), ammonia (NH_3_), 2,3-butanediol, acetoin, dimethyl disulfide (DMDS), and 1-undecene, has been reported (Howell et al., 1988; Ryu et al., 2003; Haas and Défago, 2005; Afsharmanesh et al., 2006; Farag et al., 2006; Kai and Piechulla, 2009; Kai et al., 2009, 2010; Blom et al., 2011; Meldau et al., 2013; Weise et al., 2013; Hunziker et al., 2015).

Based on the above consideration, it seemed worthy of interest to confirm the production of volatiles by *P. tolaasii* and establish their identity and biological role on the host mushroom and plants. In this work we report a study on the bioactivity of volatile compounds, produced by three virulent strains of *P. tolaasii*, on *P. eryngii* and *P. ostreatus* mycelia and *A. bisporus* and *P. ostreatus* basidiome tissue blocks. Some of the pure VOCs, identified by GC–MS, were *in vitro* evaluated for their toxicity, respectively, on the above mycelia growth and tissue blocks. Furthermore, since *P. tolaasii* is also a natural rhizosphere inhabitant of some plants, in the present study is also reported the bioactivity of both natural volatile mixture and pure VOCs on lettuce and broccoli seeds germination and seedling growth.

## Materials and Methods

### Bacterial, Fungal Cultures, and Seeds

Bacterial type strain NCPPB2192 and the strains USB1 (ICMP13791), USB66 (ICMP13792) of *P. tolaasii*, and fungal strains DS256, DS270 of *P. eryngii* and DS226, DS284 of *P. ostreatus*, have been used in this study. Bacterial strains were maintained lyophilised at 4°C and subcultures were obtained on King’s B agar medium (KBA; King et al., 1954) for 48 h at 25°C. *P. ostreatus* and *P. eryngii* strains were maintained on Malt Extract Agar (MEA, Oxoid, Milan, Italy) slants at 4°C and subcultures were obtained by growing the fungi on the same medium at 25°C for 72 h.

Broccoli (*Brassica oleracea* L. var. *italica*; variety “Cima di rapa sessantina”, Blumen, Milan, Italy) and lettuce (*Lactuca sativa* L.; variety “Lattuga romana lentissima a montare”, Blumen, Milan, Italy) seeds were used for the evaluation of bioactivity of bacterial volatiles on plant material.

### Mushroom Bioassays

Mycelia of *P. eryngii* and *P. ostreatus* strains, grown as reported above, and tissue blocks of *A. bisporus* and *P. ostreatus*, obtained from freshly harvested basidiomes, prepared according to Ercolani (1970), were used.

The assay was performed inoculating 100 μl of about 10^8^ CFU ml^-1^ (OD_590_ = 0.2) bacterial suspension of *P. tolaasii* strains on KBA in each of two out of three sectors Petri dishes. In the third sector a *Pleurotus* sp. mycelium plug (6 mm diameter) was placed on 5 ml of MEA. In the case of basidiome tissue assays, one tissue block of *A. bisporus* or *P. ostreatus* was placed onto the plate bottom. After inoculation, Petri dishes were incubated at 25°C for 5 days and then mycelia growth was measured (colony diameter subtracted the mycelium agar plug diameter) and the effects on tissue blocks were visually evaluated.

The assays as above described were performed either sealing Petri dishes with parafilm (Pechiney Plastic Packaging Company, Chicago, IL, USA) to minimize volatile compounds exchange during the incubation period or in non-sealed Petri dishes to simulate what happens in nature in an open system such as the rhizosphere.

Controls were obtained placing mycelium plugs or tissue blocks as above, but no bacterial suspensions were inoculated on KBA in the remaining Petri dishes sectors. All mushroom bioassays were performed twice with three replicates and, in the case of mycelia growth assays, the final data were reported in percentage compared to the control (100%).

### Seed Bioassays

Broccoli and lettuce seeds were surface disinfected by treating for 1 min with 0.5% chlorine, washed three times with sterile distilled water and then dried under an air flow cabinet at room temperature for 20 min. One hundred disinfected seeds were transferred on filter paper soaked with 1.5 ml of sterile distilled water in one out of three sectors of Petri dishes. The other two sectors, containing each 5 ml of KBA, were inoculated with 100 μl of about 10^8^ CFU ml^-1^ bacterial suspension of *P. tolaasii* strains. Petri dishes containing 100 broccoli or lettuce seeds in one out three sectors, while in the other two just KBA were used as controls. The assay was performed as above both in parafilm-sealed and non-sealed dishes. After 5 days incubation at 25°C in the dark, broccoli and lettuce seeds germination was assessed and the whole seedlings, epicotyls, main rootlets were measured with a ruler. The seed bioassays were performed at least twice with three replicates and the final growth data were reported as percentage compared to the control growth (100%).

### GC–MS Analysis

The three strains of *P. tolaasii*, plus *P. eryngii* strain DS256 and *P. ostreatus* strain DS284, were analyzed for their volatile compounds profile after a 5 days incubation period both in sealed and non-sealed conditions. The bacterial volatile analysis was carried out in Petri plates, prepared as reported in the previous paragraph, but without fungal inoculum or seeds, respectively, while the fungal volatile analysis was carried out in Petri plates prepared as reported in the mushroom bioassays, but without bacterial inoculum. Petri dishes with non-inoculated KBA and MEA were used as negative control.

Volatiles produced by bacteria and fungi were collected with SPME fiber, coated with 100 μm of polydimethylsiloxane (PDMS) phase (Supelco 57300-U, mounted on a Supelco 57330 support). The fiber was introduced in the headspace of Petri dishes through a hole previously obtained by piercing the parafilm layers, and kept inside the plate for 20 min. The fiber was then introduced into the injection port of a HP6890 plus gas-chromatograph equipped with a Phenomenex Zebron ZB-5 MS capillary column (30 m × 0.25 mm ID × 0.25 μm film thickness). A HP5973 mass selective detector (mass range: 15–300 amu; scan rate: 1.9 scans s^-1^; EM voltage: 1435) with helium at 0.8 ml min^-1^ as carrier gas was used. The injection port, equipped with glass insert (internal diameter 0.75 mm) was split less at 250°C. The desorption time of 1.0 min was used. Detector was maintained at 230°C. Oven was maintained at 40°C for 2 min, then the temperature increased to 250°C (8°C min^-1^) which was maintained for 10 min. All the analysis were performed twice with three replicates. A blank run was performed after each analysis in order to check for residual compounds contaminating the fiber.

All the peaks were identified by comparison with spectra in Wiley6N and NIST98 libraries. Furthermore, the identity of some of the VOCs components was confirmed by GC–MS analysis of reference substances [acetaldehyde (Sigma–Aldrich, 402788); methanethiol (MT) (Sigma–Aldrich, 295515); DMDS (Sigma–Aldrich, W353604); *p*-cymene (Sigma–Aldrich, C121452); 1-undecene (Sigma–Aldrich, 242527); 2-undecanone (Sigma–Aldrich, U1303)] used as control. The volatile relative concentrations in each Petri plate were calculated based on GC–MS peak areas without using correction factors. The results were reported as percentage average of total peak area (±SE).

### NH_3_ and HCN Production

Strains of *P. tolaasii* were screened for NH_3_ production for which freshly grown cultures were grown in 10 ml peptone water (peptone 10 g l^-1^, NaCl 5 g l^-1^, pH 7.2) tubes for 72 h at 25°C. After incubation period 0.5 ml of Nessler’s reagent [0.09 mol l^-1^ solution of K_2_(HgI_4_) in 2.5 mol l^-1^ of KOH] was added in each tube. The development of brownish color of bacterial liquid cultures was positive for NH_3_ production (Cappuccino and Sherman, 2010).

Hydrogen cyanide production was assessed inoculating bacteria in nutrient sucrose agar (sucrose 5 g l^-1^, yeast extract 4 g l^-1^, peptone 4 g l^-1^, beef extract 2 g l^-1^, agar 18 g l^-1^) amended of glycine (4.4 g l^-1^) Petri dishes. Before incubation a Whatman No. 1 filter paper disk, soaked with a solution of 0.5% picric acid in Na_2_CO_3_ (2%) aqueous solution was placed on the upper lid of each plate. The plates were finally incubated for 4 days at 25°C. Filter paper color switch from yellow to red-brown was positive for HCN production (Lorck, 1948).

Strains USB2106 of *P. putida* and USB2102 of *P. brassicacearum* were used as positive control for the production of NH_3_ and HCN, respectively. Non-inoculated media were used as negative controls. The assays were performed twice with three replicates.

### VOCs Bioassays

In order to evaluate VOCs bioactivity, identified by GC-MS, belonging to *P. tolaasii* strains volatile mixture, three pure VOCs, DMDS, MT, and 1-undecene (Sigma–Aldrich, Milan, Italy), selected on the basis of their detection for all the *P. tolaasii* strains, were used in mushrooms and seeds bioassays. In these bioassays *P. ostreatus* mycelium plugs, *A. bisporus* and *P. ostreatus* basidiome tissue blocks and 100 broccoli seeds, were placed in two out three sectors of Petri dishes, while in the third one were dropped or injected, according to their physical state, DMDS, 1-undecene, and MT. In particular, liquid DMDS and 1-undecene were dropped in glass slide posed in one of the three sectors; then the dishes were rapidly sealed with parafilm, to minimize volatile compound exchange, able to alter the volatile quantity introduced in the system. In the bioassay with gaseous MT, at first, the plates were pierced in correspondence of the empty sector and the hole closed with a triple layer of parafilm. Then the gas were taken with a GC syringe (Supelco, Milan, Italy) from a gas Pyrex pipette device (Microglass Heim s.r.l., Naples, Italy) and injected through the hole; then the dishes were immediately re-sealed and incubated for 5 days at 25°C in the dark. Petri dishes without the VOCs, were used as controls. Then mycelia and seedlings growth was measured with a ruler as above said and the effects on tissue blocks were visually evaluated. All bioassays were performed twice with three replicates and the final data were reported in percentage compared to the control.

### Statistical Analysis

All GC–MS analysis and bioassays data were statistically evaluated for the determination of standard errors and the latter subjected to analysis of variance (ANOVA) and *P* calculated by F test of Fisher–Snedecor. All statistical analysis was carried out using software program package SPSS version 17.0 (SPSS Inc., Chicago, IL, USA).

## Results

### Mushroom Bioassays

**FIGURE 1:**
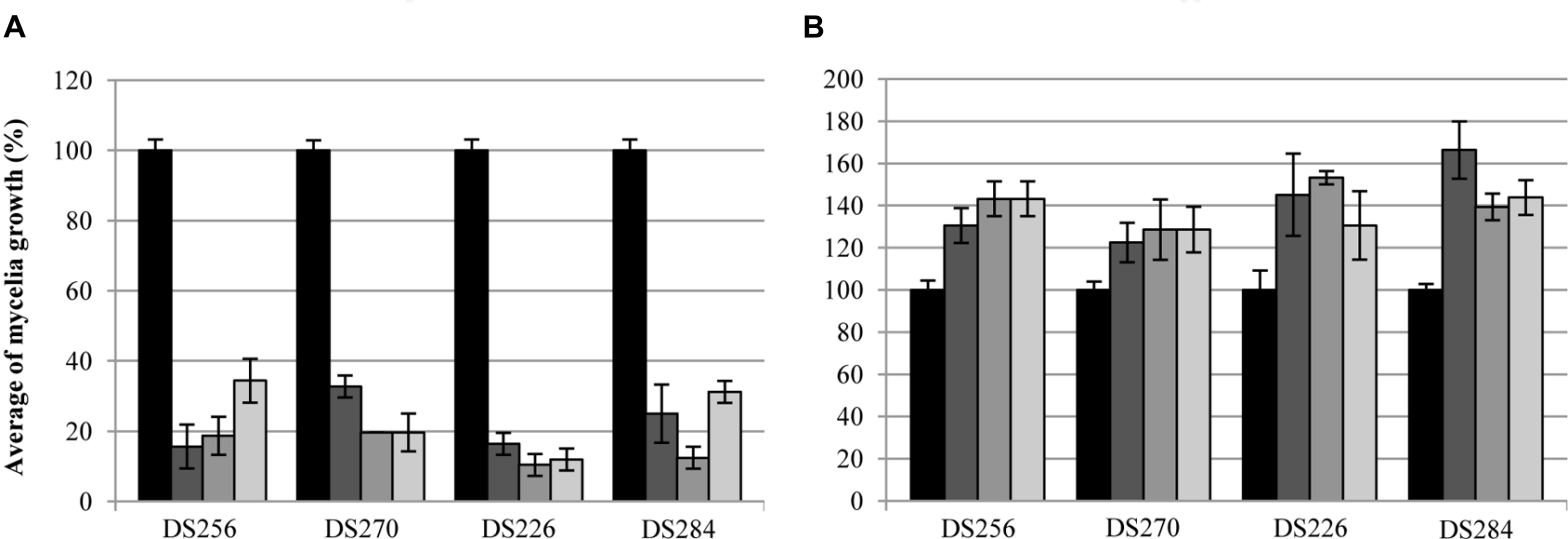
**Average of mycelia growth (%) of strains DS256, DS270, and DS226, DS284 of *Pleurotus eryngii* and *P. ostreatus*, respectively, in presence of water (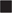) and volatile compounds produced by strains NCPPB2192 (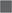), USB1 (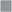), and USB66 (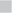) of *Pseudomonas tolaasii* in Petri dishes not sealed **(A)** and sealed **(B)** with parafilm**. Bars on the columns correspond to the standard error of the mean in percentage.

Volatile compounds produced by *P. tolaasii* strains caused a highly significant reduction, in non-sealed Petri dishes, (*P* ≤ 0.014) of *P. eryngii* and *P. ostreatus* mycelia growth (**Figure 1A**). In fact, the mycelia growth of the strains DS256 and DS270 of *P. eryngii*, in the presence of volatile compounds of the strains NCPPB2192, USB1, and USB66 of *P. tolaasii* was only 16 and 19% (NCPPB2192), 34 and 33% (USB1) and 20% for both fungi (USB66) compared to the control (100%), respectively (**Figure 1A**). In the same assay conditions, mycelia growth of the strains DS226 and DS284 of *P. ostreatus* was only 16 and 10% (NCPPB2192), 12 and 25% (USB1), 12 and 31% (USB66) of the control, respectively (**Figure 1A**).

When the bioassay was performed in sealed Petri dishes, instead, (**Figure 1B**) it came out that *P. tolaasii* strains volatiles caused a significant *P. eryngii* and *P. ostreatus* strains mycelia growth increase (*P* ≤ 0.038) (**Figure 1B**). Indeed, fungal growth of the *P. eryngii* strains DS256 and DS270, co-incubated with the *P. tolaasii* strains NCPPB2192, USB1, and USB66, was assessed to 131%, and 122% (NCPPB2192), 143 and 129% (USB1), 143 and 129% (USB66), respectively, compared to the control (**Figure 1B**). In a similar way, the effect of the above examined volatiles on mycelia growth of *P. ostreatus* strains DS226 and DS284 was 145 and 166% (NCPPB2192), 153 and 139% (USB1), 131 and 144% (USB66), respectively, compared to the control (**Figure 1B**).

**FIGURE 2:**
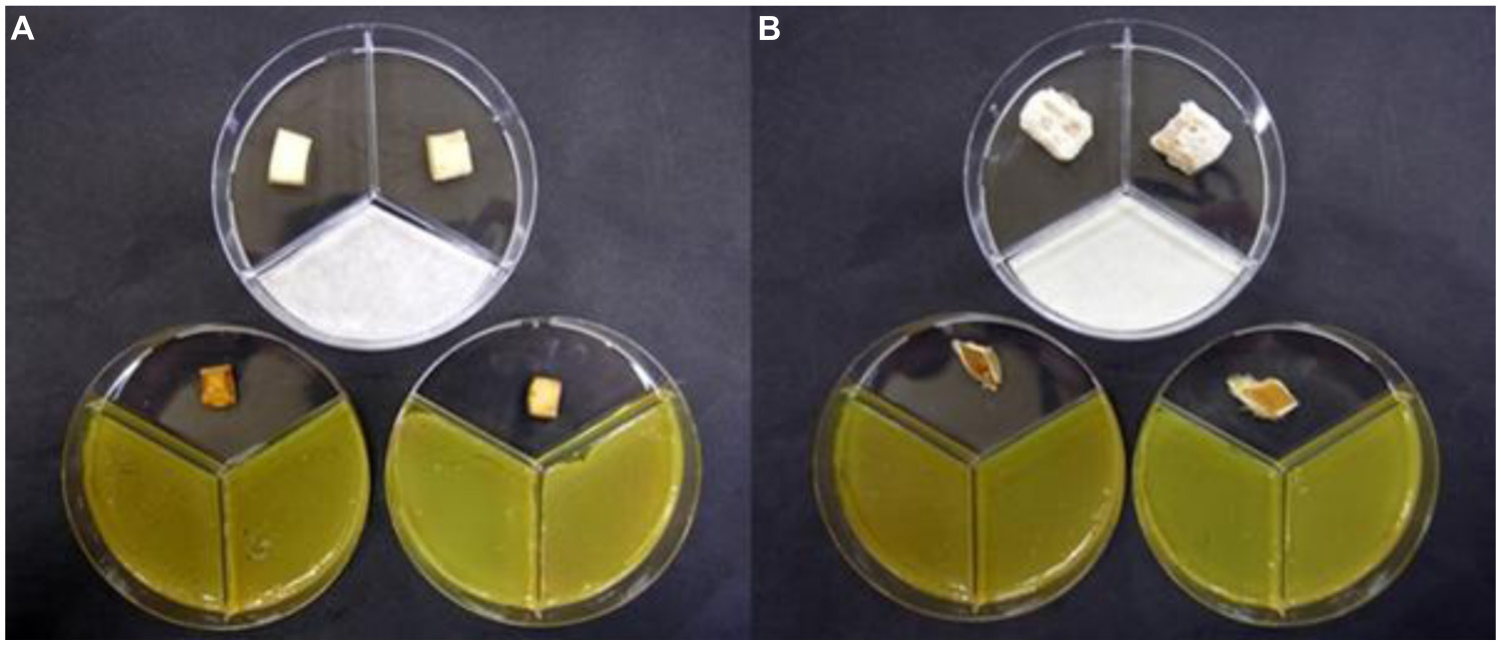
***Agaricus bisporus***(A)** and *Pleurotus ostreatus***(B)** basidiome tissue blocks showing marked brown discoloration caused by volatile compounds blend produced by *Pseudomonas tolaasii* NCPPB2192 when the Petri dishes were not sealed with parafilm**. At the top the Petri dishes containing the blocks treated with water.

**FIGURE 3:**
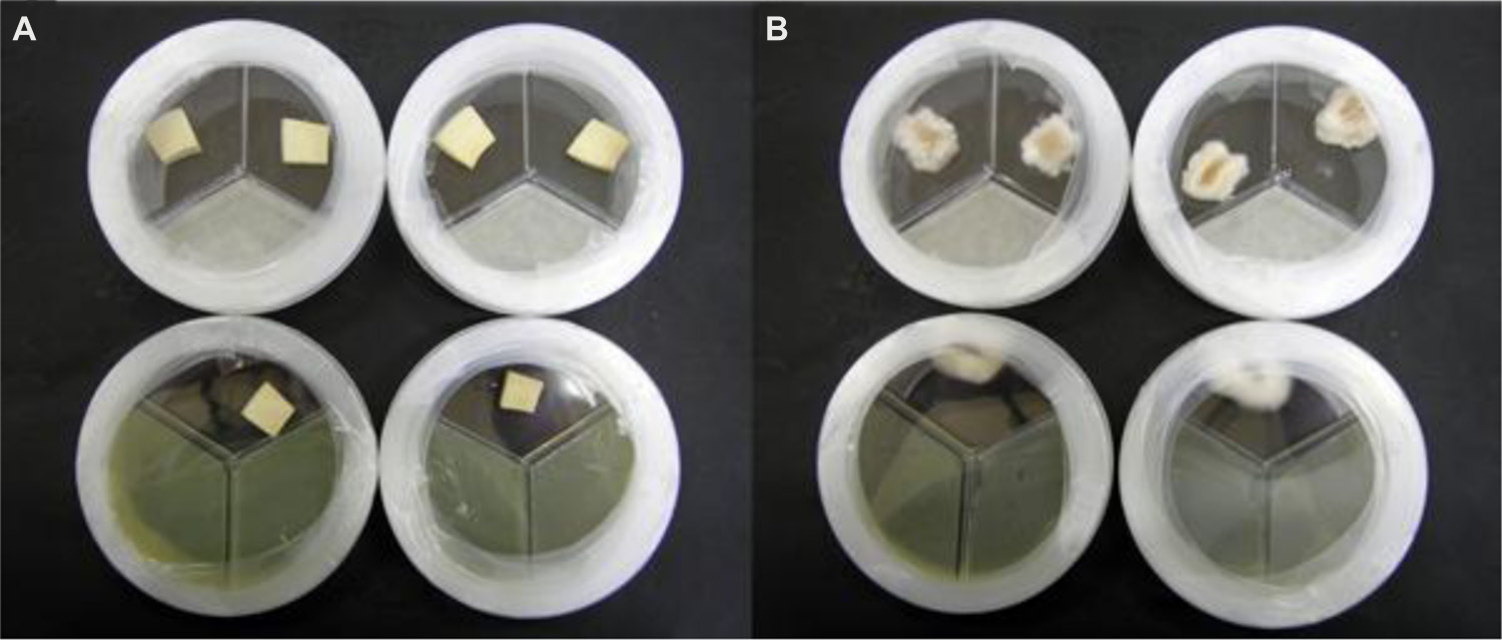
***Agaricus bisporus***(A)** and *Pleurotus ostreatus***(B)** basidiome tissue blocks showing apparently no change **(A)** or a slight increase in mycelia growth **(B)** when they were exposed, in sealed Petri dishes, to volatile compounds blend produced by *Pseudomonas tolaasii* NCPPB2192**. At the top the Petri dishes containing the blocks treated with water.

In non-sealed Petri dishes, volatiles from *P. tolaasii* strains caused a noticeable brown discoloration of *A. bisporus* and *P. ostreatus* tissue blocks (**Figure 2**); when Petri dishes were sealed, instead, no apparent alteration of the *A. bisporus* tissue blocks was noticed and the presence of mycelium growth surrounding *P. ostreatus* tissue blocks was observed (**Figure 3**).

### Seed Bioassays

**FIGURE 4:**
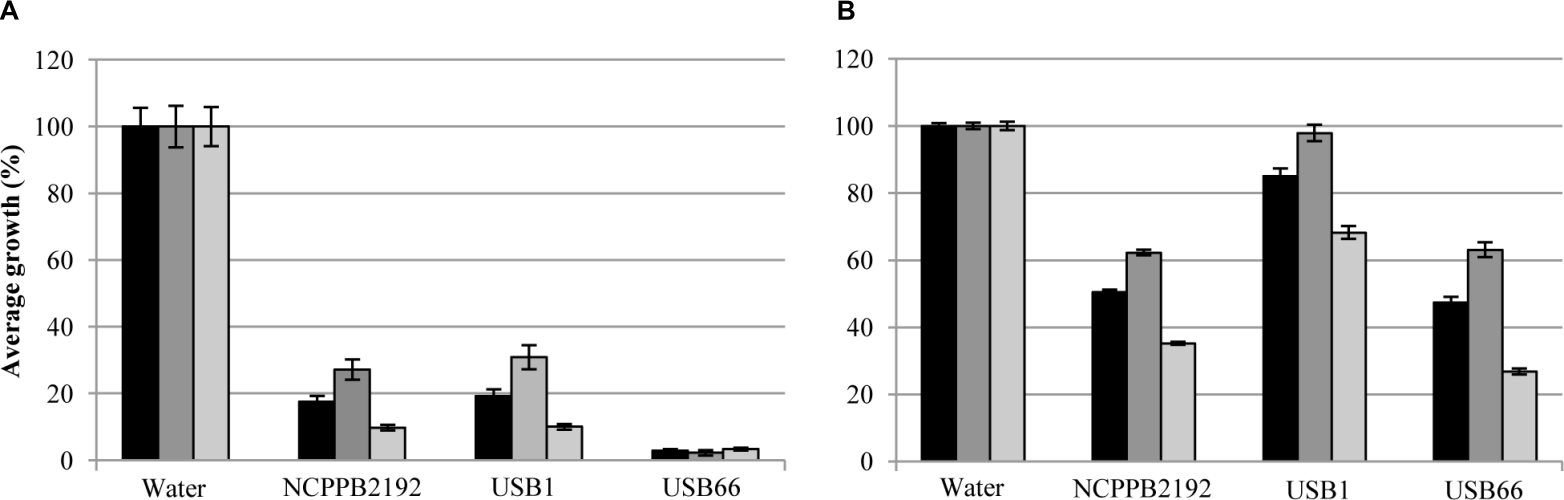
**Average growth (%) of whole seedlings 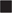), epicotyls (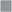), and main rootlets (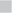) of broccoli **(A)** and lettuce **(B)** in presence of volatile compounds blend produced by strains NCPPB2192, USB1 and USB66 of *Pseudomonas tolaasii* in non-sealed Petri dishes**. Bars on the columns correspond to the standard error of the mean in percentage.

In non-sealed Petri dishes assays, volatile compounds produced by *P. tolaasii* strains NCPPB2192, USB1 and USB66 caused a highly significant reduction (*P* < 0.001) of broccoli seedlings growth, compared to the water control (**Figure 4A**). Indeed, the growth of whole seedlings, epicotyls and main rootlets was highly reduced, resulting in 17, 27, and 10% (NCPPB2192), 19, 31, and 10% (USB1), 3, 2, and 3% (USB66), respectively, compared to the control (100%) (**Figure 4A**). The above bioassays on lettuce seeds produced a similar toxic effect, though more limited. In fact, the growth of whole seedlings, epicotyls and main rootlets was highly reduced (*P* < 0.001) to 51, 62, 35, 47, 63, and 27 in presence of the NCPPB2192 and USB66 strains, respectively, compared to the control (**Figure 4B**). When the USB1 strain was used, the whole seedlings and epicotyls length of the plantlets (85 and 98%, respectively) resulted not significantly (*P* > 0.05) different from the controls, while the main rootlets length resulted highly reduced (*P* < 0.001) at 68% compared to the control (**Figure 4B**).

**FIGURE 5:**
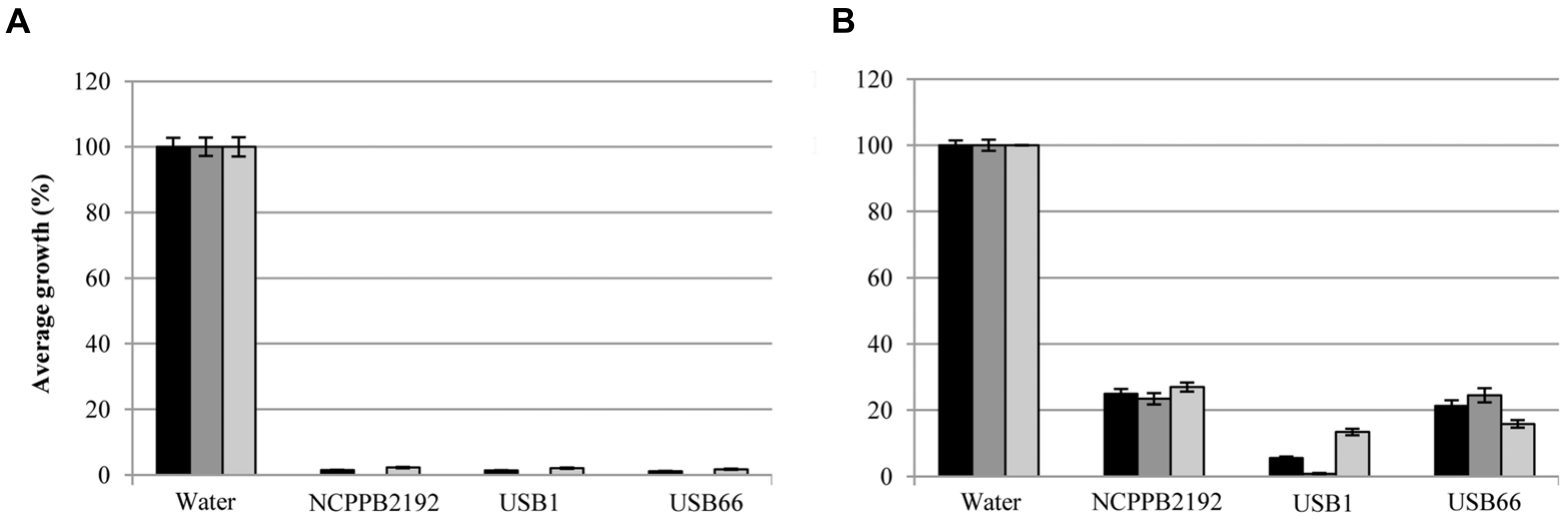
**Average growth (%) of whole seedlings (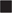), epicotyls (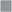), and main rootlets (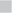) of broccoli **(A)** and lettuce **(B)** in presence of volatile compounds blend produced by strains NCPPB2192, USB1 and USB66 of *Pseudomonas tolaasii* in sealed Petri dishes**. Bars on the columns correspond to the standard error of the mean in percentage.

In sealed Petri dishes assays, the reduction of broccoli and lettuce plantlets growth was more pronounced (**Figure 5**) and the statistical analysis confirmed that volatile compounds from the above bacterial strains led to a highly significant reduction (*P* < 0.001) of broccoli and lettuce seedlings growth in comparison to the control (**Figure 5**). Actually, the growth of the whole broccoli seedlings, epicotyls and main rootlets resulted highly reduced being only 1, 0, 2% for all *P. tolaasii* strains, compared to the control (**Figure 5A**). Similarly, the growth of the whole lettuce seedlings, epicotyls, and main rootlets was 25, 23, 27% (NCPPB2192), 6, 1, 13% (USB1), and 21, 24, 16% (USB66), respectively, compared to the control (**Figure 5B**).

**FIGURE 6:**
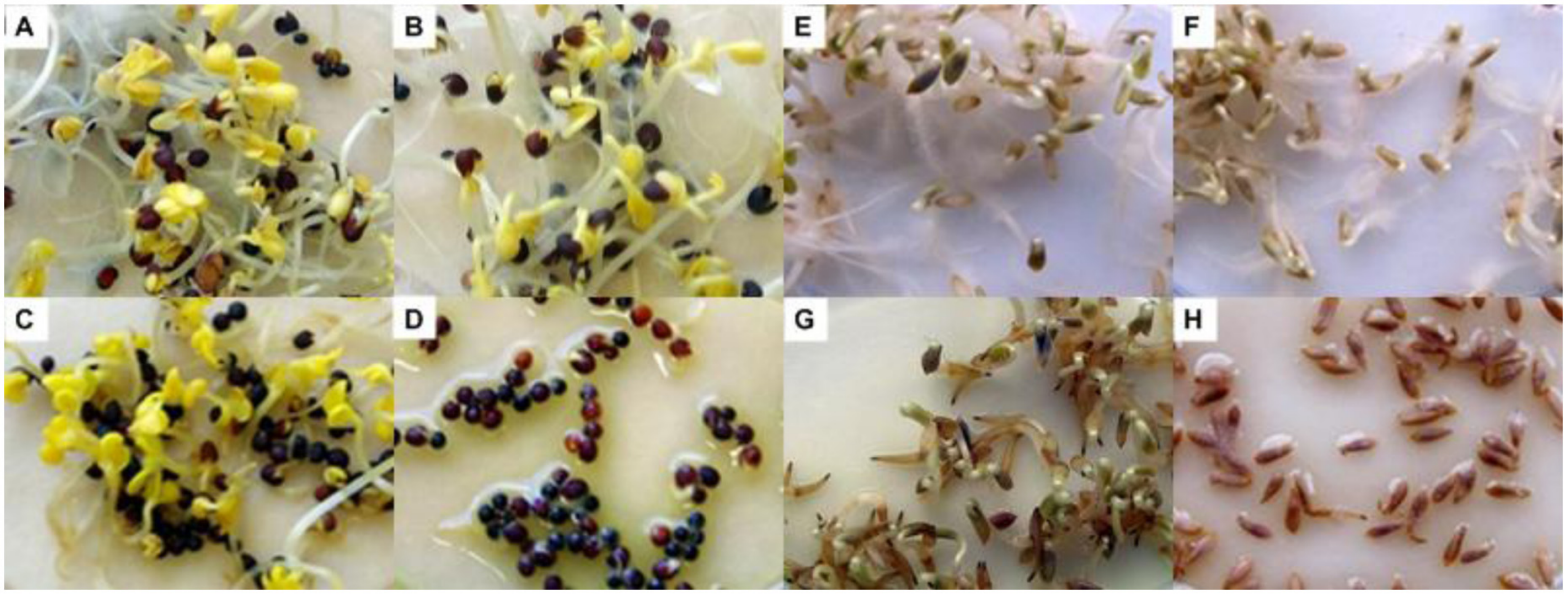
**Growth of broccoli **(A–D)** and lettuce **(E–H)** seedlings in absence (**A,B,E,F**, respectively) and in presence (**C,D,G,H**, respectively) of volatile compounds produced by strain NCPPB2192 of *Pseudomonas tolaasii* in Petri dishes not sealed **(A,C,E,G)** and sealed **(B,D,F,H)** with parafilm**.

Furthermore, the inspection of broccoli and lettuce seedlings made evident root browning and, in the case of lettuce, the absence of root hairs, as it was observed in the negative control (**Figure 6**).

### GC–MS Analysis and NH_3_ and HCN Production

**FIGURE 7:**
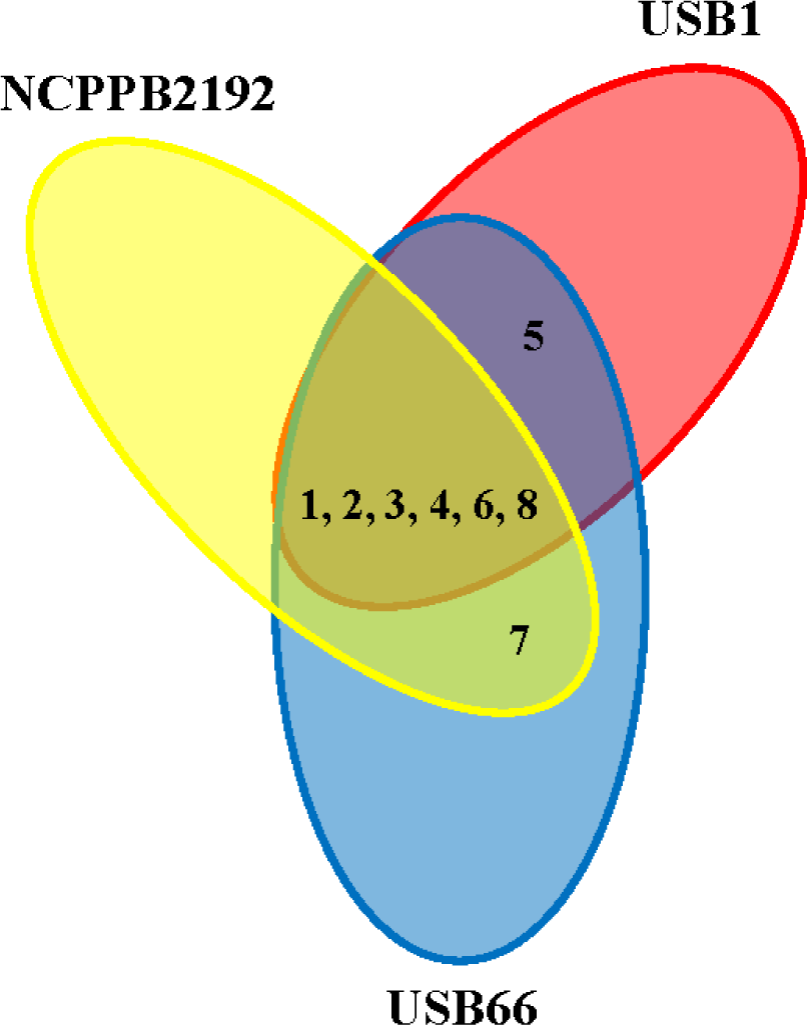
**Volatile compounds produced by *Pseudomonas tolaasii* strains (NCPPB2192, USB1, and USB66)**. 1- carbon dioxide (CO_2_); 2- methanethiol (MT); 3- dimethyl disulfide (DMDS); 4- *p*-cymene; 5, 1,4-undecadiene; 6- 1-undecene; 7- 2-undecanone; 8- 4,7-dimethylundecane.

*Pseudomonas tolaasii* strains, in sealed Petri dishes, produced MT, DMDS, *p*-cymene, 1,4-undecadiene, 1-undecene, 2-undecanone, and 4,7-dimethylundecane, even though not all the volatile compounds identified have been systematically produced by the three *P. tolaasii* strains (**Table 1**; **Figure 7**). In addition, CO_2_ was also present in the headspace of these samples. The strains DS256 and DS284, respectively, of *P. eryngii* and *P. ostreatus*, in the same experimental conditions, produced CO_2_ and nitrous oxide (NO_2_) and CO_2_, ethylene, and acetaldehyde, respectively (**Table 1**). In the headspace of plates containing non-inoculated KBA or MEA media, CO_2_, acetaldehyde, and propane, and CO_2_ and ethylene oxide, respectively, were detected (**Table 1**).

**Table 1:** Percentage average of total peak area (±SE) of volatile compounds in the overhead space of sealed Petri dishes, as determined by SPME–GC analysis, produced by pure cultures of *Pseudomonas tolaasii*, *Pleurotus eryngii*, and *P. ostreatus* strains and by non-inoculated KBA and MEA media.

Volatile compounds^a^	*P. tolaasii* strains^b^			*P. eryngii* DS256^b^	*P. ostreatus* DS284^b^	Media	
	NCPPB 2192	USB1	USB66			KB^c^	MEA^d^
Carbon dioxide (CO_2_)	63.66 ± 2.20	44.71 ± 5.22	42.73 ± 4.34	69.86 ± 1.26	6.75 ± 0.88	5.04 ± 0.22	5.31 ± 0.59
Nitrous oxide (NO_2_)	–	–	–	1.10 ± 0.11	–	–	–
Ethylene oxide	–	–	–	–	5.41 ± 0.32	–	45.25 ± 1.94
Acetaldehyde	–	–	–	–	69.42 ± 2.44	52.55 ± 1.98	–
Propane	–	–	–	–	–	2.68 ± 0.32	–
Methanethiol (MT)	2.12 ± 0.19	1.89 ± 0.19	2.03 ± 0.27	–	–	–	–
Dimethyl disulfide (DMDS)	0.25 ± 0.04	0.22 ± 0.04	0.26 ± 0.04	–	–	–	–
*p*-cymene	Traces	0.11 ± 0.04	0.10 ± 0.03	–	–	–	–
1,4-undecadiene	–	0.49 ± 0.18	0.20 ± 0.04	–	–	–	–
1-undecene	11.86 ± 2.34	17.04 ± 2.73	14.45 ± 2.08	–	–	–	–
2-undecanone	1.98 ± 0.71	–	0.40 ± 0.04	–	–	–	–
4,7-dimethylundecane	Traces	0.13 ± 0.04	0.15 ± 0.03	–	–	–	–

*^a^Volatile compounds are reported according to their molecular weight. ^b^*Pseudomonas tolaasii*, *Pleurotus eryngii*, and *P. ostreatus* strains were grown on KB and MEA, respectively; NCPPB, National Collection of Plant Pathogenic Bacteria; USB, Microorganism collection of Università (U) degli Studi (S) della Basilicata (B). ^c^KB, medium B of King et al. (1954). ^d^MEA, Malt Extract Agar. –, no volatile compounds detected*.

In non-sealed conditions no volatiles were detected.

The three *P. tolaasii* strains resulted to produce NH_3_ but not HCN.

### VOCs Bioactivity

#### Mushroom Bioassays

**FIGURE 8:**
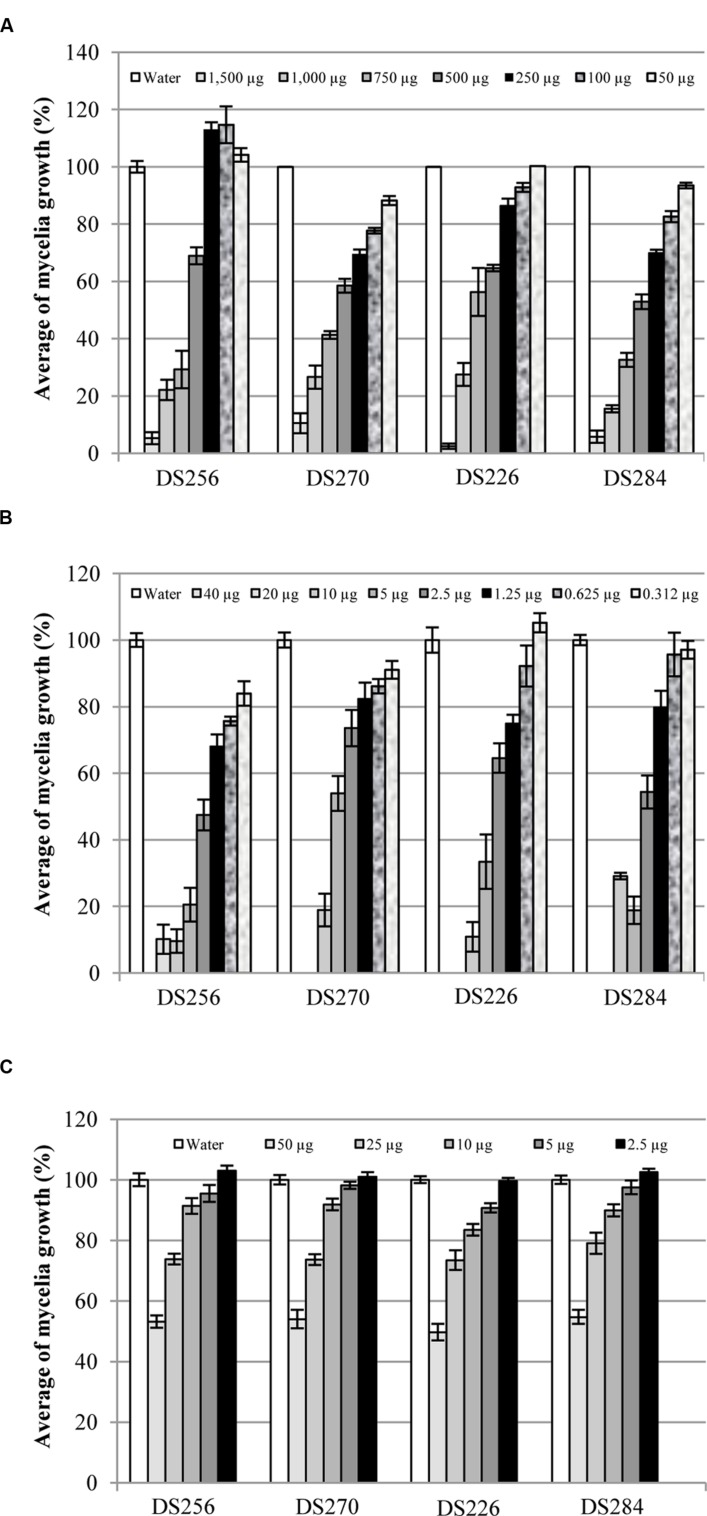
**Average of mycelia growth (%) of strains DS256, DS270, and DS226, DS284 of *Pleurotus eryngii* and *P. ostreatus*, respectively, in presence of pure MT **(A)**, DMDS **(B)**, and 1-undecene **(C)** aliquots**. Bars on the columns correspond to the standard error of the mean in percentage.

Treatments with 1,500 μg of MT per Petri dish caused a noticeable inhibition of *P. eryngii* and *P. ostreatus* strains mycelia growth (**Figure 8A**). In particular, *P. eryngii* strain DS256 resulted less sensitive than DS270 strain to MT effects. Indeed, DS256 strain growth was significantly reduced (*P* ≤ 0.001) by doses ≥500 μg [mycelia growth ≤69% when compared to the control (100%)], below which no significant growth reduction values were observed. *P. eryngii* strain DS270 resulted more affected by MT inhibiting action; using doses ≥50 μg mycelia growth was ≤88% (*P* = 0.01). The diverse sensitivity between two strains of the same mushroom was noticed also in the case of *P. ostreatus* strains DS226 and DS284, where quantities ≥500 μg of MT were necessary to cause significant growth inhibition of DS226 (mycelia growth ≤65%, *P* ≤ 0.001), and ≥100 μg for DS284 strain (mycelia growth ≤87%, *P* = 0.001) (**Figure 8A**).

The total inhibition of *P. eryngii* and *P. ostreatus* strains mycelia growth was observed when they were treated with doses ≥20 μg per Petri dish of DMDS (**Figure 8B**). At lower doses of DMDS, the effect on mycelia growth was reduced but still highly significant. In particular, at doses ≥0.625 μg per Petri dish mycelia growth of the strains DS256 and DS270 of *P. eryngii* was ≤76% (*P* = 0.013) and ≤86% (*P* = 0.002), respectively, when compared to the control. A similar effect on *P. ostreatus* DS226 and DS284 strains growth was observed applying doses ≥1.25 μg of DMDS per Petri dish. Indeed, mycelia growth of the latter strains was ≤75% (*P* = 0.001) and ≤80% (*P* ≤ 0.002), respectively, compared to the control (**Figure 8B**).

1-undecene treatments on *P. eryngii* strains did not affect mycelia growth at doses ≤10 μg while, above the mentioned quantity, it caused a significant reduction (*P* ≤ 0.001) (**Figure 8C**). In particular, *P. eryngii* mycelia growth at doses ≥25 μg was ≤74% for both *P. eryngii* strains compared to the control (**Figure 8C**). The same treatments on *P. ostreatus* strains DS226 and DS284 were ineffective in mycelia growth inhibition at doses ≤2.5 and ≤5 μg, respectively; on the contrary, using quantity ≥5 and ≥10 μg, respectively, 1-undecene caused a significant reduction of mycelia growth (≤91%, *P* = 0.002 and ≤90%, *P* = 0.038) (**Figure 8C**).

**FIGURE 9:**
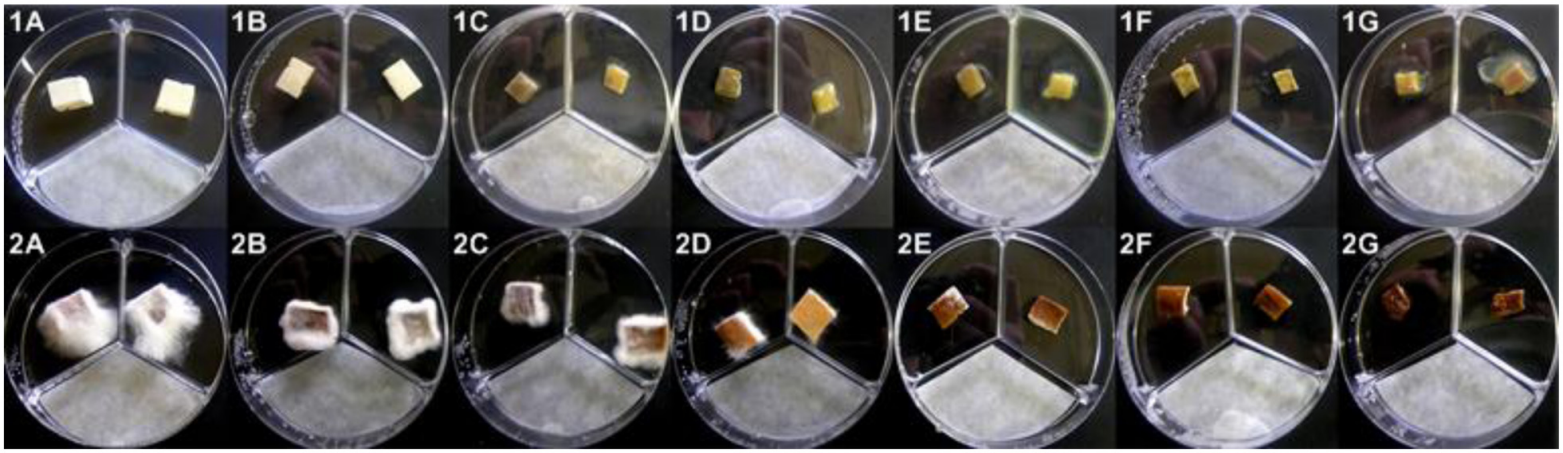
***Agaricus bisporus***(1)** and *Pleurotus ostreatus***(2)** basidiome tissue blocks treated with pure MT aliquots (**1A and 2A** = H_2_O; **1B and 2B** = 50 μg; **1C and 2C** = 100 μg; **1D and 2D** = 250 μg; **1E and 2E** = 500 μg; **1F** = 750 μg; **1G and 2F** = 1,000 μg; **2G** = 1,500 μg)**.

On *A. bisporus* and *P. ostreatus* tissue blocks MT aliquots determined aerial mycelia growth reduction, brown discoloration and deliquescence, with *P. ostreatus* being the less sensitive (**Figure 9**). Brown discoloration (**Figure 9-1C**) and tissue deliquescence of *A. bisporus* tissue blocks were determined with treatments of 100 and ≥250 μg of MT per Petri dish, respectively (**Figure 9-1D**). When *P. ostreatus* tissue blocks were treated with 50 or 100 μg of MT, the reduction of aerial mycelia growth was solely observed (**Figure 9-2B,C**); doses ≥250 μg caused a marked reduction of the mycelia growth and depressed brown discoloration (**Figure 9-2D,E**). The deliquescence of *P. ostreatus* tissue blocks was observed with treatments ≥1500 μg of MT (**Figure 9-2G**).

**FIGURE 10:**
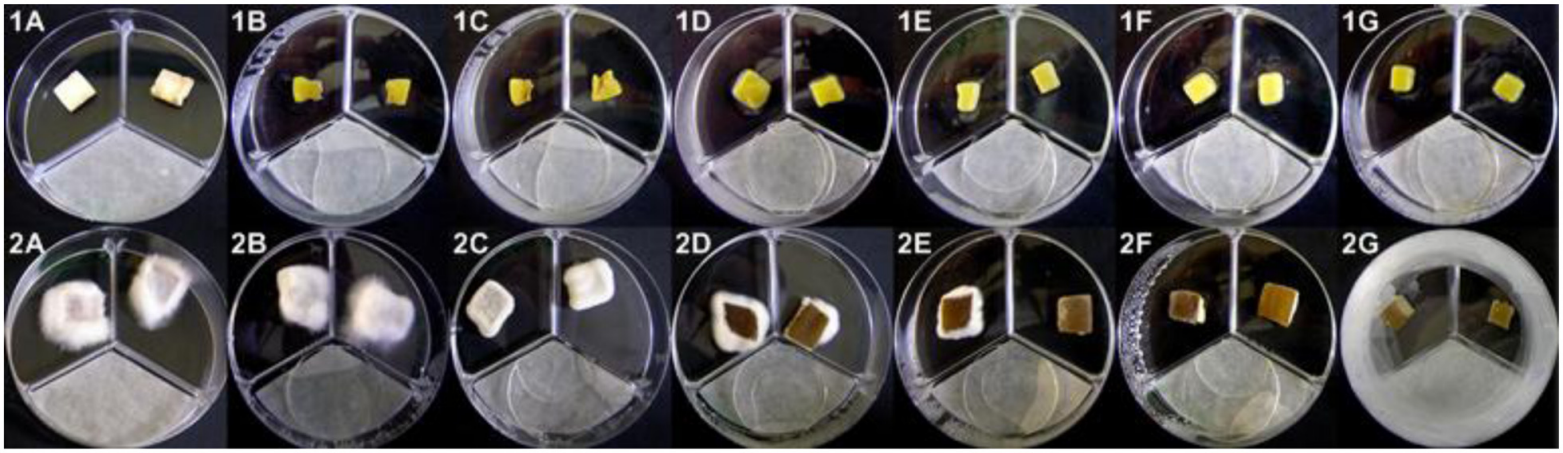
***Agaricus bisporus***(1)** and *Pleurotus ostreatus***(2)** basidiome tissue blocks treated with pure DMDS aliquots (**1A and 2A** = H_2_O; **1B** = 0.156 μg; **1C** = 0.312 μg; **1D and 2B** = 0.625 μg; **1E and 2C** = 1.25 μg; **1F and 2D** = 2.5 μg; **1G and 2E** = 5 μg; **2F** = 10 μg; **2G** = 20 μg)**.

Aerial mycelia growth reduction, yellowing, brown discoloration and deliquescence of *A. bisporus* and *P. ostreatus* tissue blocks were observed when they were treated with DMDS aliquots, even though *P. ostreatus* tissue resulted, also in this case, less sensitive than *A. bisporus* (**Figure 10**). The yellowing of *A. bisporus* tissue blocks was observed with 0.156 μg of DMDS per Petri dish treatment (**Figure 10-1B**), while the tissue deliquescence was observed with doses ≥0.625 μg (**Figure 10-1D**). Treatments with 1.25 μg of DMDS caused, on *P. ostreatus* tissue blocks, a reduction of the aerial mycelia growth (**Figure 10-2C**), whereas 2.5 μg caused brown discoloration (**Figure 10-2D**). A marked depressed brown discoloration and deliquescence of tissue was observed with doses of DMDS ≥20 μg (**Figure 10-2G**).

**FIGURE 11:**
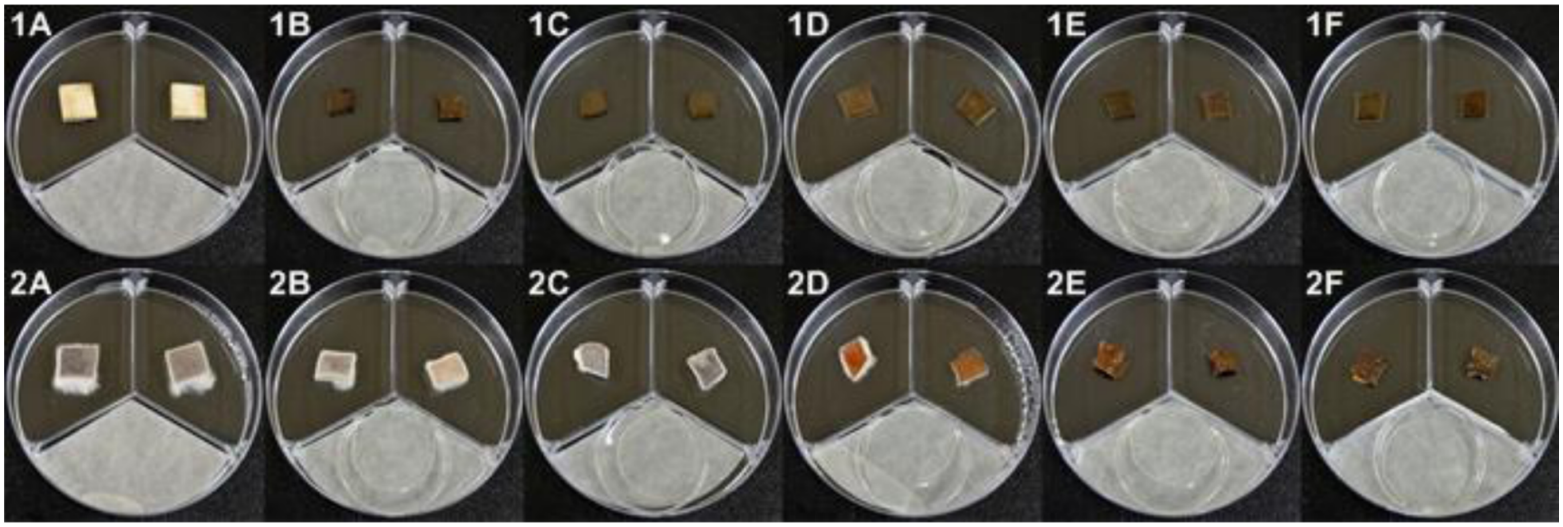
***Agaricus bisporus***(1)** and *Pleurotus ostreatus***(2)** basidiome tissue blocks treated with pure 1-undecene aliquots (**1A and 2A** = H_2_O; **1B and 2B** = 2.5 μg; **1C and 2C** = 5 μg; **1D and 2D** = 10 μg; **1E and 2E** = 25 μg; **1F and 2F** = 50 μg)**.

1-undecene treatments also caused aerial mycelia growth reduction, yellowing, brown discoloration, and deliquescence of *A. bisporus* and *P. ostreatus* tissue blocks and, in this case too, *P. ostreatus* tissue was found less sensitive than *A. bisporus* (**Figure 11**). In fact, *A. bisporus* tissue blocks showed marked depressed brown discoloration and deliquescence of tissue at all 1-undecene doses used in this work (**Figure 11-1**). On the other side, *P. ostreatus* tissue blocks at doses ≤5 μg of the VOC caused aerial mycelia growth reduction (**Figure 11-2B,C**), at doses of 10 μg determined brown discoloration (**Figure 11-2D**), and at doses ≥25 μg induced a marked depressed brown discoloration and deliquescence of tissue (**Figure 11-2E,F**).

#### Seed Bioassays

**FIGURE 12:**
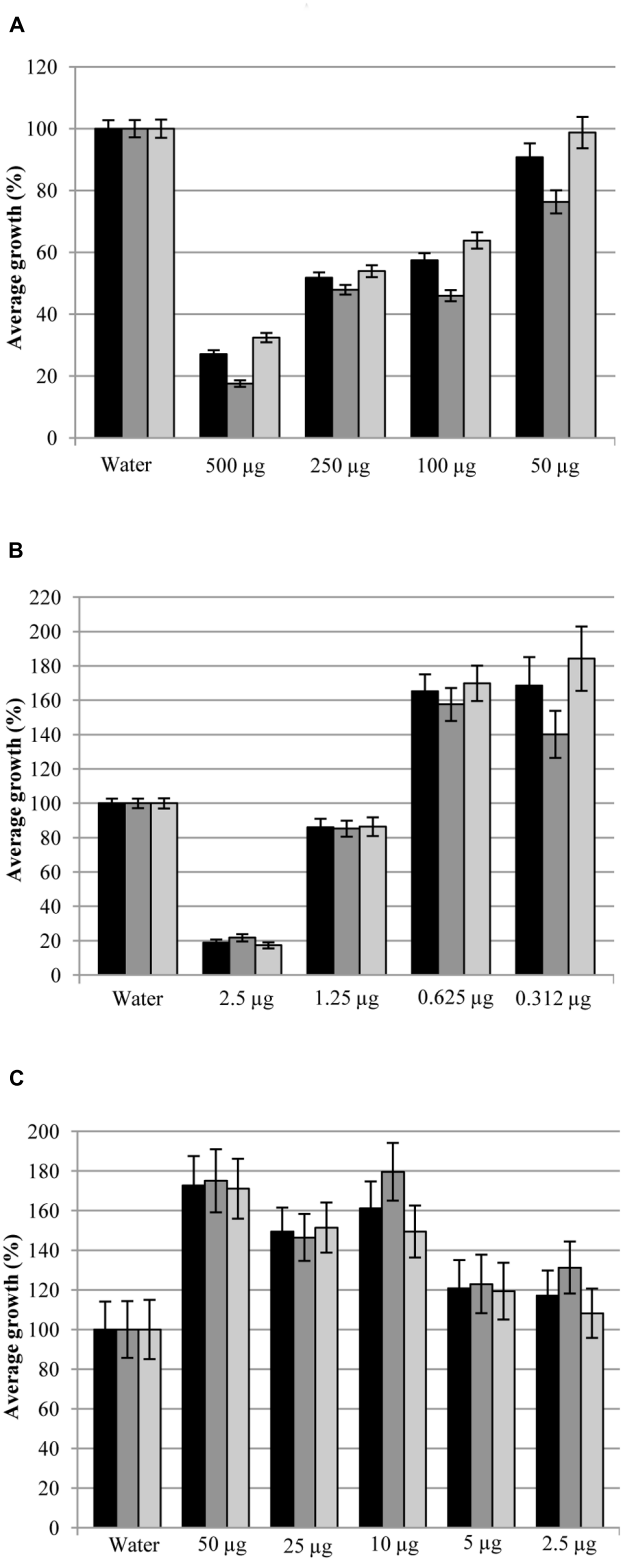
**Average growth (%) of whole seedlings (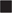), epicotyls (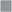), and main rootlets (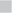) of broccoli in presence of pure MT **(A)**, DMDS **(B)**, and 1-undecene **(C)** aliquots**. Bars on the columns correspond to the standard error of the mean in percentage.

Methanethiol and DMDS at doses >500 and 5 μg per Petri dish, respectively, inhibited broccoli seeds germination. At lower doses, different effects on seedlings growth were observed (**Figure 12**). Doses of 500, 250, and 100 μg of MT led to highly significant reduction (*P* < 0.001) of whole seedling (27, 52, and 57%), epicotyl (18, 48, and 46%), and main rootlet growth (32, 54, and 64%), respectively, when compared to the control (100%) (**Figure 12A**). Treatment with 50 μg of MT caused a significant reduction (*P* ≤ 0.002) of the whole seedling (9%) and epicotyl (24%) growth but the main rootlet growth was not significantly different (99%) from the control (**Figure 12A**).

Significant reduction (*P* < 0.001) of whole seedling (19%), epicotyl (22%) and main rootlet (17%) growth, in comparison with water seed treatment (100%) used as control (**Figure 12B**), was observed after seeds treatment with 2.5 μg of DMDS. Doses of 1.25 μg of DMDS caused significant decrease (*P* < 0.001) of the whole seedling (86%) and epicotyl length (85%) while the main rootlet growth (86%) was not statistically different (*P* > 0.05) from the control (**Figure 12B**). Highly significant increase (*P* ≤ 0.002) of seedlings growth (whole seedling, epicotyl, and main rootlet) was obtained by treatments with 0.625 μg (165, 158, and 170%, respectively) and 0.312 μg (169, 140, and 184%, respectively) of DMDS, compared to the control (**Figure 12B**).

Broccoli seedlings growth stimulation was also noticed in 1-undecene treatments (**Figure 12C**). In fact, significant increase of whole seedlings, epicotyls and main rootlets growth was caused by treatments with 10 μg (161, 180, and 149%, respectively, with *P* ≤ 0.013), 25 μg (150, 147, and 151%, respectively, with *P* ≤ 0.021), and 50 μg (173, 175, and 171%, respectively, with *P* ≤ 0.05) of 1-undecene, compared to the control (**Figure 12C**).

## Discussion

In this paper, it was demonstrated that *P. tolaasii*, an ubiquitous bacterium mainly recognized as a mushroom pathogen but also reported as associated to plant in either parasitic or commensal positions (Rainey et al., 1991; Zdor and Anderson, 1992; Noval et al., 1993; Shirata et al., 1995; Talame and Piccirillo, 1995; Tanprasert and Reed, 1997), is able to produce *in vitro* volatiles which were identified by GC–MS. They were demonstrated to affect mushrooms, seed germination, and seedling growth features confirming previous evidences (Shirata, 1996). Indeed, in assays performed in non-sealed Petri dishes, *P. eryngii* and *P. ostreatus* strains mycelia growth was greatly reduced when exposed to *P. tolaasii* strains volatile metabolites. In the same experimental conditions *P. tolaasii* volatiles toxicity was explicated by pronounced *A. bisporus* and *P. ostreatus* basidiome tissue blocks brown lesions or yellowing. On the other side, *P. tolaasii* volatiles in sealed Petri dishes, did not have toxic effect: *P. eryngii* and *P. ostreatus* mycelia showed a significant growth increase and *A. bisporus* and *P. ostreatus* blocks presented even mycelia proliferation on their surfaces. These apparently divergent and contradictory results may be explained as follows. In *in vitro* experimental systems performed, there is the strictly aerobic bacterium *P. tolaasii* and *Agaricus* and *Pleurotus* sp. which are able to live also in limited oxygen conditions (Rast and Bachofen, 1967a,b; San Antonio and Thomas, 1972; Zadražil, 1975). In fact, in the latter condition *Pleurotus* sp. mycelia increase their biomasses because of their ability to assimilate CO_2_ when available in the environment at high concentrations (Rast and Bachofen, 1967a,b; San Antonio and Thomas, 1972; Zadražil, 1975). This seems the case of the sealed Petri dishes assay conditions. On the other hand the oxygen limitation may lead the bacterium to produce VOCs, which levels may be under the toxicity threshold; it could also be that the simultaneous accumulation of CO_2_, produced by both organisms, may counteract VOCs toxic effects (Rast and Bachofen, 1967a,b; San Antonio and Thomas, 1972; Zadražil, 1975).

*Pseudomonas tolaasii*, when grown in non-sealed Petri dishes, expresses its aerobic metabolism and inorganic and organic volatile compounds are produced better than the bacterium could do in limited oxygen conditions (sealed plates) (Ferchichi et al., 1986). In such state, despite the fact that part of the volatile compounds produced may be dispersed outside of the Petri dish, the VOCs still reach toxic concentrations for *Pleurotus* sp. mycelia and *A. bisporus* and *P. ostreatus* basidiome tissue blocks.

Lettuce and broccoli seeds exposure to bacterial volatiles led to negative effect on both germination and seedling growth. The highest effects were observed when the assays were carried out in sealed Petri dishes. In non-sealed dishes, with normal oxygen concentrations and gas exchange, seeds germination is just partly inhibited due to the possible sub-lethal volatiles dose achievement. In sealed plates system, the toxic effects of volatiles, since there is a progressive decrease of oxygen concentration and an accumulation of gaseous catabolites, considering also that both aerobic organisms are stressed in this conditions, may depend on possible additional/synergistic action of volatiles (Segal and Starkey, 1969; Ferchichi et al., 1986; van Leerdam et al., 2008). Moreover, the above results suggest roots as being the most sensitive plantlet part to the *P. tolaasii* toxic action volatiles since they showed some brown-necrotic areas beside growth reduction. Furthermore, despite the fact that *P. tolaasii* produced high quantities of CO_2_ and it is known that this compound is able to stimulate plant growth (Kai and Piechulla, 2009), no beneficial effect on plant biostimulation was observed.

The GC–MS analysis, despite the fact that in non-sealed conditions no volatiles were detected probably because of their dispersion, indicated that *P. tolaasii* strains are able to produce different volatiles, among which MT, DMDS, and 1-undecene have been produced by all the three strains of the bacterium. Moreover, GC–MS analysis of non-inoculated KBA or MEA and *Pleurotus* sp. strains inoculated on MEA clearly demonstrated that no one of the above mentioned volatiles were detected, confirming their exclusive origin from *P. tolaasii.* The same GC-MS analysis stated, as expected, the presence of a quite important level of CO_2_ produced by the growing microorganisms.

Finally, all *P. tolaasii* strains did not produce HCN, although this substance is produced by several bacteria including some *Pseudomonas* sp. inhabiting plants rhizosphere (Blom et al., 2011; Weise et al., 2013). However, *P. tolaasii* strains produce NH_3_ and this volatile compound has a significant role on the plants and fungi growth (Mikeš et al., 1994; Weise et al., 2013). In fact, it is known that NH_3_ inhibits the growth of *Arabidopsis thaliana* through the alkalization of the neighboring plant medium and, on the other hand, it stimulates the fungal growth via its assimilation by the glutamine synthetase and glutamate dehydrogenase NAD-dependent enzymes (Mikeš et al., 1994; Weise et al., 2013). This may contribute to explain the growth inhibition of broccoli and lettuce seedlings and increased mycelia growth in biological assays performed in this study.

On the basis of these results the VOCs MT, DMDS, and 1-undecene were selected for further assays.

The *in vitro* assays results, performed using pure VOCs showed that MT, DMDS, and 1-undecene, whose toxic action on pathogenic fungi is well known (Lewis, 1985; Fritsch, 2005; Groenhagen et al., 2013; Wang et al., 2013; Popova et al., 2014; Hunziker et al., 2015), are also toxic for the *P. eryngii* and *P. ostreatus* mycelia and *A. bisporus* and *P. ostreatus* tissue blocks. Furthermore, higher MT and DMDS doses (from 50 to 500 μg and ≥1.25 μg, respectively) inhibited the broccoli seeds germination while the DMDS and 1-undecene (at doses ≤0.625 and ≥10 μg, respectively) have also caused an increase of whole broccoli seedlings growth. The results concerning the bioactivity of the above mentioned VOCs and, in particular, on plant growth stimulation action by 1-undecene have here been reported, to the best of our knowledge, for the first time.

These findings clearly indicate that MT and DMDS have an important role in the bioactivity of bacterial volatiles natural blend toward fungal and plant systems. The toxic effect of these VOCs may have cellular respiration as target, since it is known that MT and DMDS are able to inhibit mitochondrial activity (Waller, 1977; Dugravot et al., 2003). Our recent electron microscopy studies (Giorgio et al., 2015) revealed that DMDS and other VOCs, such as 2-nonanone and DL-limonene, are also able to determine structural alterations of cell membranes on phytopathogenic fungi that can contribute, along with the alteration of mitochondrial activity, to cell death. DMDS also proved capable, at doses ≤0.625 μg, of stimulating the growth broccoli seedlings. These results are not very different from the ones obtained by other authors. In fact, Kai et al. (2009) showed that DMDS inhibits *A. thaliana* (L.) Heynh growth and Groenhagen et al. (2013) have highlighted that the same molecule, at doses ranging from 1 ng to 1 mg, significantly increased the same plant biomass. Recently, Meldau et al. (2013) demonstrated that DMDS is able to make available its organic sulfur to the roots of *Nicotiana attenuata* Torr. ex S. Watson favoring its growth.

1-undecene’s ability to inhibit *Pleurotus* sp. strains growth, to alter *A. bisporus* and *P. ostreatus* blocks and increase the growth of broccoli seedlings are, to our knowledge, new biological properties for this compound. Antimicrobial activity of 1-undecene has been only recently determined (Kai et al., 2009; Groenhagen et al., 2013; Wang et al., 2013; Popova et al., 2014) on some target microorganisms [*Agrobacterium tumefaciens* Smith & Townsend, *Synechococcus* sp., *Rhizoctonia solani* (Cooke) Wint. and *Fusarium culmorum* (Wm. G. Sm.) Sacc.] among which only the fungus *F. culmorum* was weakly inhibited; biostimulation ability on plants of this molecule, on the other side, remains totally unexplored. Some authors (Nisenbaum et al., 2013) have previously shown that hydrocarbons, including 1-undecene, can be assimilated or transformed by microorganisms, but to our knowledge, no one has proven whether broccoli or plants in general, are able to utilize 1-undecene as carbon source. This outcome certainly highlights the need for further studies to understand this result.

## Conclusion

*Pseudomonas tolaasii* volatile blend, as well as pure VOCs used in the present work, may have an important role in the interaction between pathogen and cultivated mushrooms. Shirata (1996) reported that *P. tolaasii* volatiles were able to contribute to the disease symptoms and interfere with mushrooms mycelia development, during substrate colonization, and thus, indirectly on mushrooms production. The fact that MT, DMDS and 1-undecene showed antifungal activity toward cultivated mushrooms mycelia and were toxic toward basidiome tissue blocks suggest the mentioned VOCs as *P. tolaasii* new potential virulent factors since they were able to reproduce, at least in part, brown blotch and yellowing typical symptoms caused by bacteria on *A. bisporus* and *P. ostreatus*, respectively. However, it is necessary to consider that, in some of the case, the above VOCs reproduced the biological effect of the whole volatile blend when applied in high, probably non-natural, concentrations. New studies would unravel whether the bacterium is able to produce *in vivo* VOCs among resulting toxic to mushrooms and if VOCs synergic actions may occur. *P. tolaasii* mutants availability in producing VOCs may highlight their contribution, beside tolaasins, on the virulence of the pathogen. Moreover, since *P. tolaasii* is also reported to be associated to plants rhizospheres (Zdor and Anderson, 1992), it is not to be excluded that VOCs, possibly produced in those niches, may interfere with the behavior of organisms dwelling the same habitat. In this regard, *P. tolaasii*, could present typical biocontrol agents traits since it is able to produce lipodepsipeptides and VOCs which are toxic for fungi. Studies to verify the rhizosphere competence of the bacterium would be very useful to assess his candidacy as a biocontrol agent.

Nevertheless, our results on these VOCs biological activity indicate that they may represent alternatives to methyl bromide for fumigation of soils infected by soil-borne fungal pathogens. As a matter of fact, DMDS is already used as a novel pre-planting soil fumigant under the commercial name PALADIN. Furthermore, it has been recently established the ability of DMDS to control plant pathogenic fungi (Fritsch, 2005; Kai et al., 2009), either directly or via the induction of systemic resistance (Huang et al., 2012), nematodes (Coosemans, 2005) and weeds (Freeman et al., 2009). Finally, since DMDS and 1-undecene were able to increase plant growth, they can also be candidate as potential plant biostimulators/fertilizers.

## Conflict of Interest Statement

The authors declare that the research was conducted in the absence of any commercial or financial relationships that could be construed as a potential conflict of interest.
